# International ranking systems for universities and institutions: a critical appraisal

**DOI:** 10.1186/1741-7015-5-30

**Published:** 2007-10-25

**Authors:** John PA Ioannidis, Nikolaos A Patsopoulos, Fotini K Kavvoura, Athina Tatsioni, Evangelos Evangelou, Ioanna Kouri, Despina G Contopoulos-Ioannidis, George Liberopoulos

**Affiliations:** 1 Department of Hygiene and Epidemiology, University of Ioannina School of Medicine, Ioannina 45110, Greece; 2 Biomedical Research Institute, Foundation for Research and Technology-Hellas, Ioannina 45110, Greece; 3 Institute for Clinical Research and Health Policy Studies, Department of Medicine, Tufts University School of Medicine, Boston, MA 02111, USA; 4 Department of Pediatrics, University of Ioannina School of Medicine, Ioannina, Greece; 5 Department of Pediatrics, George Washington University School of Medicine and Health Sciences, Washington DC, USA

## Abstract

**Background:**

Ranking of universities and institutions has attracted wide attention recently. Several systems have been proposed that attempt to rank academic institutions worldwide.

**Methods:**

We review the two most publicly visible ranking systems, the Shanghai Jiao Tong University 'Academic Ranking of World Universities' and the Times Higher Education Supplement 'World University Rankings' and also briefly review other ranking systems that use different criteria. We assess the construct validity for educational and research excellence and the measurement validity of each of the proposed ranking criteria, and try to identify generic challenges in international ranking of universities and institutions.

**Results:**

None of the reviewed criteria for international ranking seems to have very good construct validity for both educational and research excellence, and most don't have very good construct validity even for just one of these two aspects of excellence. Measurement error for many items is also considerable or is not possible to determine due to lack of publication of the relevant data and methodology details. The concordance between the 2006 rankings by Shanghai and Times is modest at best, with only 133 universities shared in their top 200 lists. The examination of the existing international ranking systems suggests that generic challenges include adjustment for institutional size, definition of institutions, implications of average measurements of excellence versus measurements of extremes, adjustments for scientific field, time frame of measurement and allocation of credit for excellence.

**Conclusion:**

Naïve lists of international institutional rankings that do not address these fundamental challenges with transparent methods are misleading and should be abandoned. We make some suggestions on how focused and standardized evaluations of excellence could be improved and placed in proper context.

## Background

The evaluation of the performance of universities and institutions is an attractive concept. In theory, objective and accurate evaluations of institutional excellence may help allocate funding rationally, prioritize research and educational investment, inform the public, guide the burgeoning market of candidate students and junior researchers, and help institutions in internal self-evaluation and improvement. International ranking of universities and institutions has received wide attention in the last few years within higher education, administrators, as well as in the broader public. In Google the words *university ranking *return 42700000 hits.

The purpose of the current manuscript is to examine critically the most popularized existing international ranking systems, assess their validity and derive insights for specific issues that need to be addressed, if international ranking of institutions is to be reliable and useful in measuring and promoting excellence. Our appraisal focuses primary (but not exclusively) on the international ranking systems that have drawn the greatest attention on the web, the Shanghai Jiao Tong University [1] 'Academic Ranking of World Universities' and the Times Higher Education Supplement [2] 'World University Rankings'. We focus on these two ranking systems because they already have a history of producing lists of institutions and they are very popular based on their appearance in web searches. In contrast to this huge public impact, there is still a dearth of peer-reviewed scientific publications on international ranking methods. Raw data and several key details about the methodology still remain unavailable to public scrutiny. We discuss issues of construct validity and measurement validity for each of the items that have been proposed as components of excellence in the ranking process. Finally, we use this information to make a list of the generic challenges that need to be met in international rankings of institutional excellence.

## Methods

### Sources of data

We focus on systems that use explicit criteria to rank universities around the world in terms of excellence, regardless of whether other institutions (e.g. non-university research institutes) are also ranked or not. The information for the discussed ranking systems is obtained from perusal of their web sites [1,2] and any associated peer-reviewed publications. We performed a search of PubMed and the Web of Science (search term 'university* AND ranking*', last search December 2006) that showed that of the two most popularized international ranking systems, only one has been described in the peer-reviewed literature [3,4] and this was only after it had already received fierce criticism [5]. No other international ranking systems have had their methods described in peer-reviewed publications as of December 2006, but we also consider briefly other systems that use different criteria, based on their web description. The concordance between the two main ranking systems was evaluated in terms of their agreement for the top 200 universities based on their publicized 2006 rankings.

### Validity assessment methods and generic issues

We assessed each of the proposed criteria for excellence in terms of construct validity and measurement validity. Construct validity refers to whether an indicator measures what it is intended to measure (i.e. excellence). We considered separately excellence in education and excellence in research. Other parameters of excellence may also matter (e.g. societal contribution, provision of healthcare), but may be even more difficult to measure. Measurement validity refers to the errors that may ensue in the measurement process.

Literature searches in the Web of Science were made focused on specific criteria to try to identify evidence that would be pertinent to the construct and measurement validity of each item. For research indicators, we used the databases of the Thomson ISI Web of Knowledge (as of December 2006), including the Web of Science, Essential Science Indicators, ISI Highly Cited, and Journal Citation Reports. Information on affiliation of Nobel Prize winners and authors of the most-cited papers was derived from the Nobel Prize website with perusal of the listed curricula vitae and autobiographies [6] and the perusal of the recent publication record in the Web of Science, respectively.

For the rating of validity for each item/criterion, we used a 4-point rating scale (poor, low/modest, good, very good) for all items. Poor means that the specific criterion is unlikely to be useful as a valid measure of excellence. Low/modest means that the specific criterion has some correlation with excellence, but this is either weak or very indirect. Good means that the specific criterion has considerable potential for capturing excellence. Very good means that the specific criterion has a strong potential for capturing excellence. We used a consensus approach for rating with iterative discussion among the authors (led by JPAI) after the evidence on the validity of each criterion had been collected and shared.

Based on the experience obtained from scrutinizing the proposed criteria and the evidence regarding their construct and measurement validity, we generated, through discussion among the authors, a list of generic issues that should be addressed in current or future efforts to rank institutions for excellence internationally.

## Results

### Description and validity of existing international rankings

#### Brief description of Shanghai and Times rankings

The Shanghai ranking [1] uses a weighted composite sum. Shanghai appraises education and faculty based on Nobel- and Fields-winning alumni/faculty and highly-cited researchers. It measures research by counting non-review articles in *Nature *and *Science*, and the total number of published articles. Also, a weighted average of these indicators is adjusted for institutional size and contributes 10% to the final sum.

The Times ranking [2] is also a composite system. The ranking assigns much weight (40% of total) to an expert opinion survey. Additional components address the rating from graduate recruiters, recruitment of international faculty, the enrollment of international students, the student to faculty ratio, and total citation counts.

#### Validity of Shanghai ranking

Nobel and Fields awards clearly measure research excellence, even if they don't cover all fields. However, it is unclear why universities with Nobel- or Fields-winning alumni are those that provide the best education. As for faculty, Nobel- and Fields-winners typically have performed their groundbreaking work elsewhere. We found that of 22 Nobel Prize winners in Medicine/Physiology in 1997–2006, only seven did their award-winning work at the institution they were affiliated with when they received the award (Table 1). Therefore, this measurement addresses the ability of institutions to attract prestigious awardees rather than being the site where groundbreaking work is performed. Finally, the vast majority of institutions have no such awardees. Thus, such criteria can rank only a few institutions.

**Table 1:** Nobel winners in Medicine/Physiology for 1997–2006: affiliation at the time they did the award-winning work and at the time they were given the Nobel Prize

**Name**	**Year**	**Affiliation (Nobel work)**	**Affiliation (Nobel award)**
Fire AZ	2006	Carnegie Institute, Washington	Stanford University
Mello CC	2006	University of Massachusetts	Same
Marshall BJ	2005	Royal Perth Hospital, Australia	University of Western Australia, Nedlands
Warren JR	2005	Royal Perth Hospital, Australia	Perth, Australia (private address)
Axel R	2004	Columbia University	Same
Buck LB	2004	Columbia University	Fred Hutchinson Cancer Research Center
Lauterbur PC	2003	SUNY Stony Brook	University of Illinois
Mansfield P	2003	University of Nottingham	Same
Brenner S	2002	MRC Molecular Biology Unit, Cambridge	Molecular Science Institute, Berkeley
Horvitz HR	2002	Cambridge University	MIT
Sulston JE	2002	MRC Molecular Biology Unit, Cambridge	Sanger Institute, Cambridge
Hartwell LH	2001	Cal Tech	Fred Hutchinson Cancer Research Center
Hunt RT	2001	Cambridge University	Imperial Cancer Research Fund, London
Nurse PM	2001	University of Edinburgh	Imperial Cancer Research Fund, London
Carlsson A	2000	University of Lund	Göteborg University
Greengard P	2000	Yale University	Rockefeller University
Kandel ER	2000	Columbia University	Same
Blobel G	1999	Rockefeller University	Same
Furchgott RF	1998	SUNY, Brooklyn	Same
Ignarro LJ	1998	Tulane University	UCLA
Murad F	1998	University of Virginia	University of Texas
Prusiner SB	1997	UCSF	Same

The determination of scientists with the highest impact has also good construct validity for research excellence, but highly-cited status has some measurement problems. It is based on a database [7] that counts raw citations. Ten citations in a single-authored paper or in a paper as, for example, 342nd author from 865 others, counts as the same [8]. There is no widely accepted alternative on how to adjust citation indices for the number of co-authors; weighting the exact contribution of an author in a paper remains elusive. The database also tries (appropriately so) to separate scientific fields, but this is unavoidably imperfect. Scientists with more multidisciplinary work have more difficulty passing the highly-cited threshold in any one field. Within the same field, scientists in sub-fields with higher citation densities have an advantage. For example, all 'Clinical Medicine' (including 1790 journals and over 1500000 author names in the last decade) [9] is treated as a single field. Approximately 250 scientists are selected per field regardless of the denominator (all authors), but there are 21 times more author names in 'Clinical Medicine' than in 'Space Science' [9]. Finally, highly-cited status is based on two decades of citations (1981–1999), a distant surrogate of current work [10]. We found that among the corresponding authors of the 10 most-cited articles published as recently as 1996–1999 and 2000–2003, 5/10 and 2/10, respectively, had changed institutions or were deceased by 2006 (Table 2) [9].

**Table 2:** Corresponding authors of the 10 most-cited papers published in 1996–1999 and the 10 most-cited papers published in 2000–2003 (citations as of end of 2006)

**Name**	**Year**	**Affiliation (most-cited paper)**	**Current affiliation**
Altschul SF	1997	NLM/NCBI	Same
Otwinowski Z	1997	University of Texas	Same
Brunger AT	1998	Yale University	Stanford University
Jeanmougin F	1997	IGBMC, INSERM	No publications 1998-present day
Ross R	1999	University of Washington	Deceased
Perdew JP	1996	Tulane University	Same
Banchereau J	1998	Baylor Research Institute	Baylor Institute for Immunology Research*
Kalnay E	1996	NCEP	University of Maryland
Posada D	1998	Brigham Young University	University of Vigo
Botstein D	1998	Stanford University	Same
Lander ES	2001	Whitehead Institute for Biomedical Research	MIT**
Berman HM	2000	Rutgers University	Same
Cleerman JI	2001	NHLBI	Same
Venter JC	2001	Celera Genomics	J Craig Venter Institute
Hanahan D	2000	UCSF	Same
Roussouw JE	2002	NHLBI	Same
Spek AL	2003	University of Utrecht	Same
Spergel DN	2003	Princeton University	Same
Tuschl T	2001	Max Planck Institute	University of Basel/Rockefeller University
Kumar S	2001	Arizona State University	Same

*No change in affiliation; change in the name of the same institution.

**No change in affiliations, but change in preference for which affiliation is listed more prominently in Essential Science Indicators records.

Counting the names and affiliations of authors in each non-review paper in *Nature *and *Science *is easy and carries negligible measurement error. Construct validity is more problematic. Overall, these two journals publish 22% of the most-cited articles across all scientific fields, but this varies from 54% in 'Immunology' to less than 7% in eight of a total of 21 scientific fields [11]. Moreover, reviews are often more-cited than any 'original' article [12,13] and their exclusion may not be justified.

Finally, the number of articles is influenced by the database used, and says nothing about their impact [14]. Rewarding the publication of more papers regardless of impact may end up reinforcing bulk science, salami publication and least publishable unit practices [15,16].

#### Validity of Times ranking

If properly performed, most scientists would consider peer review to have very good construct validity; many may even consider it the gold standard for appraising excellence. However, even peers need some standardized input data to peer review. The Times simply asks each expert to list the 30 universities they regard as top institutions of their area without offering input data on any performance indicators. Research products may occasionally be more visible to outsiders, but it is unlikely that any expert possesses a global view of the inner workings of teaching at institutions worldwide. Moreover, the expert selection process of The Times is entirely unclear. The survey response rate among the selected experts was only <1% in 2006 (1600 of 190000 contacted). In the absence of any guarantee for protection from selection biases, measurement validity can be very problematic. The opinion of graduate recruiters probably has poor construct validity for academic excellence, while it does measure the market impact of education; measurements are provided by a sample of 736 recruiters with undisclosed response rate and selection process.

The international character of an institution is an interesting aspect, but its construct validity for determining excellence is unknown. International character probably reflects resource, administrative and legislation issues. Institutions offering competitive packages may recruit more international faculty from those with limited resources. International faculty and student enrollment may also be dictated largely by local or national regulations (e.g. allowed teaching languages). Enrollment of students from foreign countries in particular may often reflect the tuition system or the wealth of recruited international students (e.g. if foreigners pay higher fees) rather than true diversity, let alone excellence. In general, student applications and recruitment are determined by a complex array of factors that only distally reflect excellence [17], and sometimes they may be negatively correlated with excellence in research (e.g. at least one study in Canada has found that high research output of a university discourages student applications [18]). The optimal student to faculty ratio is difficult to generalize across different disciplines and settings. Finally, the quality of the measurements for such international data is also not transparent.

The total number of citations has much better construct validity for addressing scientific impact than total number of papers. Even though citations are not always reflective of approval of a scientific work, they do reflect its contribution to scientific debate. However, one should carefully adjust for scientific field. Moreover, differences across citation databases, errors in automated citation counts [19], self-citation, different citation rates across scientific fields [14], and non-standardized handling of group authorship papers [20], pose some measurement error limitations. Using citation databases requires careful cleaning of the raw data and there is no hint that Times undertakes any such cleaning.

### Agreement in international rankings

**Figure 1:**
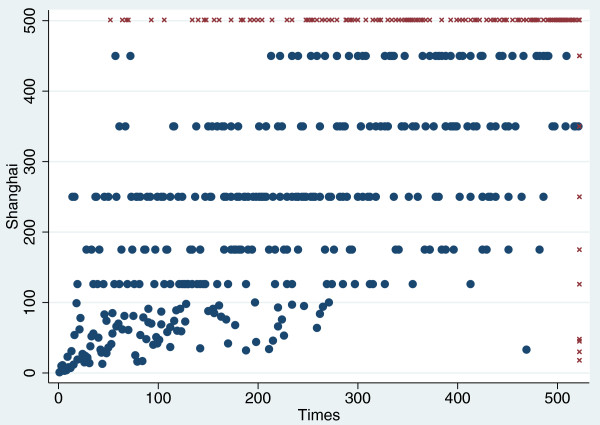
**Correlation between Shanghai and Times ranking systems**. Data are considered for the top 500 universities in the Shanghai and Times systems. Cross marks denote universities ranked outside illustrated rank positions in either system. Note that for Shanghai it is common for several universities to have the same aggregate score and thus share the same rank (the median value of the span of ranks involved).

In 206, the Shanghai and Times lists shared only 133 universities among their top 200s. Some discrepancies are notorious (Figure 1): four of the top 50 on the Shanghai list did not even make the top 500 of the Times list, and several top Times choices disappeared on the Shanghai list (Table 3). Some of the discrepancies reflect the fact that The Times does not consider institutions that have no undergraduate education (e.g. UCSF and Rockefeller). However, discrepancies extend well beyond this difference (Table 3). As both systems claim to measure institutional excellence (even with different indicators), the lack of better concordance is disquieting.

**Table 3:** Examples of marked discrepancies in Shanghai vs Times rankings

**Institution**	**Rank**
Institutions in the top 70 of the Shanghai list not making the top 500 of the Times list	
University of California San Francisco	Shanghai rank = 18
Rockefeller University	Shanghai rank = 30
Universite Paris 06	Shanghai rank = 45
Karolinska Institutet	Shanghai rank = 48
Institutions in the top 70 of the Times list not making the top 500 of the Shanghai list	
Fondation des Sciences Politiques	Times rank = 52
Ecole Polytech Fed Lausanne	Times rank = 64
Indian Institutes of Management	Times rank = 68
School of Oriental and African Studies	Times rank = 70

### Other options

A brief discussion of some other rankings may offer additional insights. Some ranking systems evaluate institutional web presence [21]. However, web connectivity does not necessarily reflect educational or research excellence, and it is search-engine dependent. Moreover, any effort to appraise the relevance, quality, source, or purpose of web links is difficult. At best, cyber-presence is an experimental ranking method.

Institutions are also ranked on research funding. This is more popular for national-level rankings, e.g. in Canada 'RESEARCH Infosource' publicizes a list whose highlight is 'The $100 Million Club' [22]. Comparisons of institutions in countries with different opportunities are unfair and different disciplines attract very different funding [23]. Even within the same country, high funding could actually signal low quality, if not accompanied by proportional achievements. A fundamental question is whether funding is a means to a goal or the goal itself. In addition, attribution of funding entails decisions on whether funding to affiliated hospitals or research institutes should be attributed to the main institution/university, whether all sources of funding should count or just competitive sources, and how to count collaborative multi-institutional grants.

Hybrid systems have also emerged. Newsweek [24] published its own set, largely amalgamating the Times and Shanghai rankings. Such high-visibility hybrids prove the attractiveness of ranking exercises, but also their glaring sloppiness. Table 4 summarizes the extent of problems in construct and measurement validity for various components of the systems discussed above.

**Table 4:** Construct validity for excellence and measurement validity of discussed ranking systems

	**Construct validity for excellence**		**Measurement validity**
	**Research**	**Education**	

**Shanghai**			
Alumni, Nobel/Fields	-	-	++
Faculty, Nobel/Fields	+++	+	++
Faculty, highly-cited	++	+	+
Nature/Science articles	++	-	+++
Number of articles	-	-	+
Size	-	-	-
**Times**			
Peer opinion	+++	+++	-
Graduate recruiter opinion	-	+	-
International faculty	+	+	?
International students	-	+	?
Student-faculty ratio	-	+	?
Citations per faculty	++	-	+
**Other rankings**			
Web presence	+	+	+
Funding	+	-	+

-, Poor; +, low/modest; ++, good; +++, very good; ?, unknown (insufficient detail provided on the reliability of databases).

### Generic issues in institutional rankings

#### Adjustment for size

Most of the ranking indicators discussed above depend on institution size. Larger institutions may have more papers, citations, award-winning scientists, students, web-links and funding. Size plays a minor role in the calculations used for the Shanghai and Times lists. For the Shanghai list, 10% of the weight addresses institutional size. For the Times list, only citations are adjusted for faculty numbers.

Normalization is potentially conceivable for analyses at the country level [25], where adjustments can be made for population or wealth indices that are well standardized internationally. Conversely, there are no internationally standardized data on 'size' of institutions. It is unknown how exactly the Shanghai and Times lists make adjustments (raw data are not publicly available). Even if one assembles faculty quotas worldwide, definitions differ. Definitions vary even across schools in the same university. Harvard lists 10674 medical faculty staff, but only 2497 faculty staff for all other schools combined [26]. Comparisons across institutions in different countries are tenuous. Finally, excellence is not necessarily linearly proportional to number of faculty, but may be also affected by availability of support staff and infrastructure. Redundancy, attainment of critical mass and multiplicative effects of collaboration are difficult to model.

#### Defining the institutions

Definition of the institutions to be ranked is not always straightforward. The size and nature (types of scientific fields included and their relative representation) of an institution varies depending on whether it is split or not to subunits and affiliates. For example, the University of California or the University of Illinois comprise many campuses each, and there are a large number of Max Planck Institutes. For medicine in particular, hospitals are the main components of a university, but not all hospital work originates from university faculties. Merging (or not) hospitals with their universities unavoidably changes rankings. The same applies to affiliated research institutes and spin-offs.

#### Averages and extremes

Any institution is a conglomerate of schools, departments, teams, and single scientists working in very different fields. An aggregate ranking may not do justice to the constituent parts. This is a form of the well-known ecological fallacy: the average misrepresents its components. If an institution is comprised of two departments with grades of 10/10 and 0/10, the average (5/10) grossly misrepresents both departments.

Some indices measure either the overall performance (e.g. number of papers or citations – either total or average per faculty), while others focus on the extremes of the distribution (e.g. Nobel winners, highly-cited researchers, top 1% most-cited papers). Both types of information may be useful, depending on what we want to know. Finally, the description of extremes may need to consider not only the best extremes, but also the worst extremes (e.g. researchers convicted of fraud, faculties with no or minimal citations, uncited papers).

None of the existing international ranking systems aims at quantifying the intra-institutional diversity in performance. This is a loss of significant information that would be more helpful in providing constructive feedback to institutions. Diversity becomes even larger when we consider between-scientist variability within the same institution.

#### Adjustment for field

Many indices depend on the scientific field. For example, according to the Thomson ISI classification of fields (n = 21) [9], 'Clinical Medicine' journals publish 20 times more papers that cumulatively receive 50 times more citations than 'Economics/Business' journals [9]. Only 0.15% of papers in 'Mathematics' receive over 100 citations within a decade from their publication, while this happens to 10% of papers in 'Molecular Biology' [9].

The Shanghai list recently developed a system for separate rankings in each of five fields [1]. This highlights the problems with superficial field adjustments. Grouping is arbitrary: Natural Sciences and Mathematics, Engineering/Technology and Computer Sciences, Life and Agricultural sciences, Clinical Medicine and Pharmacy, and Social Sciences; Arts and Humanities and Psychology/Psychiatry are excluded. Ranking criteria are similar to the overall ranking, with some modifications, e.g. consideration of number of articles in 'top' high-impact journals per field, instead of articles in *Nature*/*Science*. 'Top' journals are determined based on impact factors, but these are not comparable across the many disciplines amalgamated into the five larger fields. For example, the discipline of 'Agriculture, Soil Science' (highest journal impact factor 2.414) is merged into the same large field as 'Immunology' (highest journal impact factor 47.400) [9]. Moreover, the distribution of citations for articles in any journal is left-skewed, with 20% of the articles taking 80% of the citations, so impact factor is a modest correlate of specific article impact [27].

How many scientific fields are there? Each of the 21 fields of Thomson ISI [9] includes many sub-fields that are occasionally quite different among themselves. Other classifications get somehow different results. Based on citation network analyses, W Bradford Paley and colleagues recently described 23 main fields that contain 776 different scientific discipline nodes [28]. Even once we agree on how to split fields, there is still no consensus on how exactly to adjust for field in conglomerate appraisals of complex institutions.

Furthermore, in existing conglomerate rankings, institutions focusing in only one or a few fields only are under-ranked, even though this focus may be inherent in their mission. Finally, some indicators are rather meaningless for select fields (e.g. number of journal publications or journal citations for arts and humanities).

#### Measurement time frame

Many useful indices, such as citation impact, require a time distance to be determined. As we discussed above, if this time distance is long, the measurement may be largely irrelevant to the current status of an institution. This is probably less of a concern for very large institutions with long traditions. The recruitment or loss of a few influential scientists or teams will not change their overall picture much. Mathematical sociology simulations show that large groups persist for longer, if they are capable of dynamically altering their membership, while smaller groups thrive when their composition remains unchanged [29]. For the majority of smaller institutions, modest changes may have a major impact over time.

#### Credit allocation

The time frame is one of the parameters influencing what institution should get credit for what. As we showed above, credit allocation for prestigious award winners and influential scientists depends on whether we focus on where they did their work vs where they work currently. Another major issue is how to assign credit for tasks that require collaboration between multiple scientists and institutions. For example, among two equally-cited papers, a paper authored by investigators in 100 institutions counts 100 times more in the Times calculations than a paper from a single institution. There is no consensus on whether this imbalance should be corrected and, if so, how. Credit allocation is also be influenced by institutional definition (discussed above).

Table 5 summarizes the extent of problems arising from issues of average vs extremes focus, field adjustment, measurement time frame and credit allocation in Shanghai and Times.

**Table 5:** Focus on extremes of excellence vs averages, appropriate field adjustment, time frame of measurement and credit allocation problems of Shanghai and Times ranking systems

	Focus*	**Field adjustment**	**Time frame of measurement**	**Credit allocation**
**Shanghai**				
Alumni, Nobel/Fields	Very extreme excellence	Not all fields represented	Typically very remote	Problematic
Faculty, Nobel/Fields	Very extreme excellence	Not all fields represented	Typically remote	Problematic
Faculty, highly-cited	Extreme excellence	To some extent	Remote (1981–1999)	Problematic
Nature/Science articles	Extreme excellence	Uneven per field	Recent (last 5 years)	Reasonable***
Number of articles	Average excellence	None	Very recent (last year)	Reasonable***
Size	Not applicable**	None	Sources unclear	Straightforward
**Times**				
Peer opinion	Varies per expert	To some extent	Varies per expert	Varies per expert
Recruiter opinion	Not applicable**	None	Varies per recruiter	Varies per recruiter
International faculty	Not applicable**	None	Sources unclear	Straightforward
International students	Not applicable**	None	Sources unclear	Straightforward
Student-faculty ratio	Not applicable**	None	Sources unclear	Straightforward
Citations per faculty	Average excellence	None	Recent (last 5 years)	Reasonable***

*Whether excellence is appraised based on the extremes or the average of the distribution of performance.

**Indicators pertain to the whole institution, so they are average indicators, but as per Table 4 they are unlikely to be more than low/modest indicators of excellence.

***Decisions need to be made regarding allocation of credit for multi-authored papers, variable credit according to authorship position etc

## Discussion

Current international rankings reflect a naive wish to summarize in a convenient way processes that are very interesting to study, but also extremely complex. Excellence is important to define, measure, interpret and improve. However, wrong appraisals may lead to inappropriate characterizations and corrective actions. The serious limitations of these exercises should be recognized. Current international rankings seem too poor to carry serious scientific credibility.

As ranking exercises acquire influence for funding, institutions and scientists may seek to excel in the specific criteria requested for excellence. The existing ranking criteria could actually harm science and education. For the Shanghai list, most institutions will be unable to attract more Nobel and Fields awards or top highly-cited scientists or even increase their presence in *Nature *and *Science*, while inflating publication numbers with junk science is easy. For the Times list, some of the 'international character' criteria would encourage global brain drain [30]. All criteria that fail to properly adjust for institutional size favor the creation of mega-size universities with unknown consequences. Large centers of excellence may accelerate some research with the accumulation of global talent, but may drain academic institutions where they are more badly needed as vehicles for social improvement and innovation [31].

Some of the same problems exist even for country-level appraisals [32], but measurement problems are more manageable. Detailed discussion of national evaluation systems is beyond our intention. Nevertheless, for some countries, evaluation agencies accumulate relatively standardized, clean information and some also use adjustments – with the caveats discussed above. Still, several popular national ranking systems have major impact despite clearly spurious methods. One example is US News and World Reports, whose highly visible rankings have been criticized repeatedly [33-36]. Even national systems with more careful methods and meticulous (even burdensome) data collection have been attacked [18,36].

## Conclusion

Despite the failure of current international ranking systems, reliable information on specific performance indices may be useful, if properly analyzed and interpreted. In general, validity may decrease as we move from appraising single scientists, to appraising teams, departments, schools, and whole institutions, and problems are maximized when we cross national boundaries. Therefore, we think that focused appraisals of single scientists and teams should take precedence over overarching appraisals of institutions. For international institutional appraisals, information can be improved by global collaboration to standardize data on key aspects of universities and other institutions. However, remaining deficits in the quality and unavoidable inconsistencies in the definitions of the collected information should be transparently admitted and their possible impact should not be underestimated. Evaluation exercises should aim at describing accurately the existing diversity rather than force spurious averages and oversimplified rankings. All performance indices should be interpreted strictly for what they stand. Finally, as probably no measurement has perfect construct validity for the many faces of excellence, efforts to improve institutions should not focus just on the numbers being watched.

## Competing interests

The author(s) declare that they have no competing interests.

## Authors' contributions

The original idea was generated by JPAI. All authors discussed the concepts involved and the protocol for evaluations and analyses and all authors collected data. JPAI wrote the first draft of the manuscript and all authors reviewed it critically. All authors have approved the submitted version.

## Pre-publication history

The pre-publication history for this paper can be accessed here:



## References

[B1] Academic Ranking of World Universities – 2006. http://ed.sjtu.edu.cn/ranking.htm.

[B2] Education news & resources at the Times Higher Education Supplement. http://www.thes.co.uk/worldrankings/.

[B3] Cheng Y, Liu NC (2006). A first approach to the classification of the top 500 world universities by their disciplinary characteristics using scientometrics. Scientometrics.

[B4] Liu NC, Cheng Y, Liu L (2005). Academic ranking of world universities using scientometrics – a comment to the "Fatal Attraction". Scientometrics.

[B5] Van Raan AFJ (2005). Fatal attraction: conceptual and methodological problems in the ranking of universities by bibliometric methods. Scientometrics.

[B6] Nobel Prizes. http://nobelprize.org/nobel_prizes/lists/all/.

[B7] ISI highlycited.com. http://isihighlycited.com.

[B8] Slone RM (1996). Coauthors' contributions to major papers published in the AJR: frequency of undeserved coauthorship. AJR Am J Roentgenol.

[B9] Thomson Scientific Essential Science Indicators module, Web of Knowledge. http://portal.isiknowledge.com.ezproxy.library.tufts.edu/portal.cgi?DestApp=ESI&Func=Frame.

[B10] Van Raan AFJ (2006). Comparison of the Hirsch-index with standard bibliometric indicators and with peer judgment for 147 chemistry research groups. Scientometrics.

[B11] Ioannidis JP (2006). Concentration of the most-cited papers in the scientific literature: analysis of journal ecosystems. PLoS One.

[B12] Patsopoulos NA, Analatos AA, Ioannidis JP (2005). Relative citation impact of various study designs in the health sciences. JAMA.

[B13] Bhandari M, Swiontkowski MF, Einhorn TA, Tornetta P, Schemitsch EH (2004). Interobserver agreement in the application of levels of evidence to scientific papers in the American volume of the Journal of Bone and Joint Surgery. J Bone Joint Surg Am.

[B14] Garfield E (2006). The history and meaning of the journal impact factor. JAMA.

[B15] Schein M, Paladugu R (2001). Redundant surgical publications: tip of the iceberg?. Surgery.

[B16] Kostoff RN, Johnson D, Rio JA, Bloomfield LA, Shlesinger MF (2006). Duplicate publication and 'paper inflation' in the Fractals literature. Sci Eng Ethics.

[B17] Heller DE (1997). Student price response in higher education – An update to Leslie and Brinkman. J Higher Educ.

[B18] Drewes T, Michael C (2006). How do students choose a university? An analysis of applications to universities in Ontario, Canada. Res Higher Educ.

[B19] Tomlinson S (2000). The research assessment exercise and medical research. BMJ.

[B20] Dickersin K, Scherer R, Suci ES, Gil-Montero M (2002). Problems with indexing and citation of articles with group authorship. JAMA.

[B21] World Universities' ranking on the Web. http://www.webometrics.info.

[B22] Research infosources. http://www.researchinfosource.com.

[B23] Giles J (2006). Plan to rank universities fails to impress. Nature.

[B24] The Top 100 Global Universities (Newsweek: International Editions – MSNBC.com). http://www.msnbc.msn.com/id/14321230.

[B25] King DA (2004). The scientific impact of nations. Nature.

[B26] Harvard at a glance. http://www.news.harvard.edu/glance/.

[B27] Garfield E (1979). Citation Indexing : Its Theory and Application in Science, Technology and Humanities.

[B28] Map of Science. http://wbpaley.com/brad/mapOfScience/index.html.

[B29] Palla G, Barabasi AL, Vicsek T (2007). Quantifying social group evolution. Nature.

[B30] Ioannidis JP (2004). Global estimates of high-level brain drain and deficit. FASEB J.

[B31] Ioannidis JP, Ahmed T, Awasthi S, Clarfield AM, Clark J (2007). Open letter to the leader of academic medicine. BMJ.

[B32] Dill DD, Soo M (2005). Academic quality, league tables, and public policy: A cross-national analysis of university ranking systerns. Higher Educ.

[B33] Green RG, Baskind FR, Fassler A, Jordan A (2006). The validity of the 2004 U.S. News & World Report's rankings of schools of social work. Soc Work.

[B34] McGaghie WC, Thompson JA (2001). America's best medical schools: a critique of the U.S. News & World Report rankings. Acad Med.

[B35] Butler D (2007). Academics strike back at spurious rankings. Nature.

[B36] Banatvala J, Bell P, Symonds M (2005). The Research Assessment Exercise is bad for UK medicine. Lancet.

